# The ever-growing complexity of the mitochondrial fission machinery

**DOI:** 10.1007/s00018-017-2603-0

**Published:** 2017-08-05

**Authors:** Alessandro Pagliuso, Pascale Cossart, Fabrizia Stavru

**Affiliations:** 1 Unité des Interactions Bactéries-Cellules, Institut Pasteur, Paris, France; 2 U604 Inserm, Paris, France; 3 USC2020 INRA, Paris, France; 4 SNC5101 CNRS, Paris, France

**Keywords:** Mitochondrial dynamics, Drp1, Cytoskeleton, Actin, Septin, Dynamin, ER, Nucleoid, mtDNA, Dyn2, Mitochondrial fusion

## Abstract

The mitochondrial network constantly changes and remodels its shape to face the cellular energy demand. In human cells, mitochondrial fusion is regulated by the large, evolutionarily conserved GTPases Mfn1 and Mfn2, which are embedded in the mitochondrial outer membrane, and by OPA1, embedded in the mitochondrial inner membrane. In contrast, the soluble dynamin-related GTPase Drp1 is recruited from the cytosol to mitochondria and is key to mitochondrial fission. A number of new players have been recently involved in Drp1-dependent mitochondrial fission, ranging from large cellular structures such as the ER and the cytoskeleton to the surprising involvement of the endocytic dynamin 2 in the terminal abscission step. Here we review the recent findings that have expanded the mechanistic model for the mitochondrial fission process in human cells and highlight open questions.

**Electronic supplementary material:**

The online version of this article (doi:10.1007/s00018-017-2603-0) contains supplementary material, which is available to authorized users.

## Introduction

Discovered by Richard Altmann in the 1840s, mitochondria are thought to derive from the invasion of a pre-eukaryotic cell by an alphaproteobacterium of the Rickettsiales order. The monophyletic origin of mitochondria suggests that this invasion was a unique event and their advent was critical for eukaryotic evolution. This is reflected by the multitude of vital functions mitochondria are responsible for, such as energy production, the biogenesis of essential cellular components, apoptosis, or innate immune signaling. In line with this, mitochondrial dysfunction correlates with a wide variety of pathologies, in particular neurodegenerative disease and cardiac dysfunction [1].

### Mitochondria are dynamic organelles

Mitochondria are particularly complex organelles, enveloped by two membranes: the outer mitochondrial membrane (OMM) and the inner mitochondrial membrane (IMM), which enclose two compartments, the intermembrane space and the matrix. The matrix harbours the mitochondrial DNA (mtDNA), a highly reduced version of the alphaproteobacterial mitochondrial ancestor’s genome. In mammalian cells, mitochondria are not isolated, bacteria-like organelles, but rather form a dynamic network. Mitochondrial dynamics and the resulting overall morphology of the network are determined by fusion and fission events and by mitochondrial movement, both of which are highly dependent on the cell type and on the functional state of mitochondria.

Mitochondria move along cytoskeletal tracks but the extent of their motility is very variable and both species and cell type dependent [2]. For example, mitochondria appear almost immotile in muscle cells, but motility is crucial for mitochondria distribution in highly polarized cells, such as neurons, where mitochondrial activity is required at the synapse. In this context the kinesin KIF5, along with dyneins and the mitochondrial adaptors Miro and Milton, mediate mitochondria movement along microtubules in higher eukaryotic cells [3], while mitochondria movement mainly relies on actin microfilaments in budding yeast [4].

**Fig. 1:**
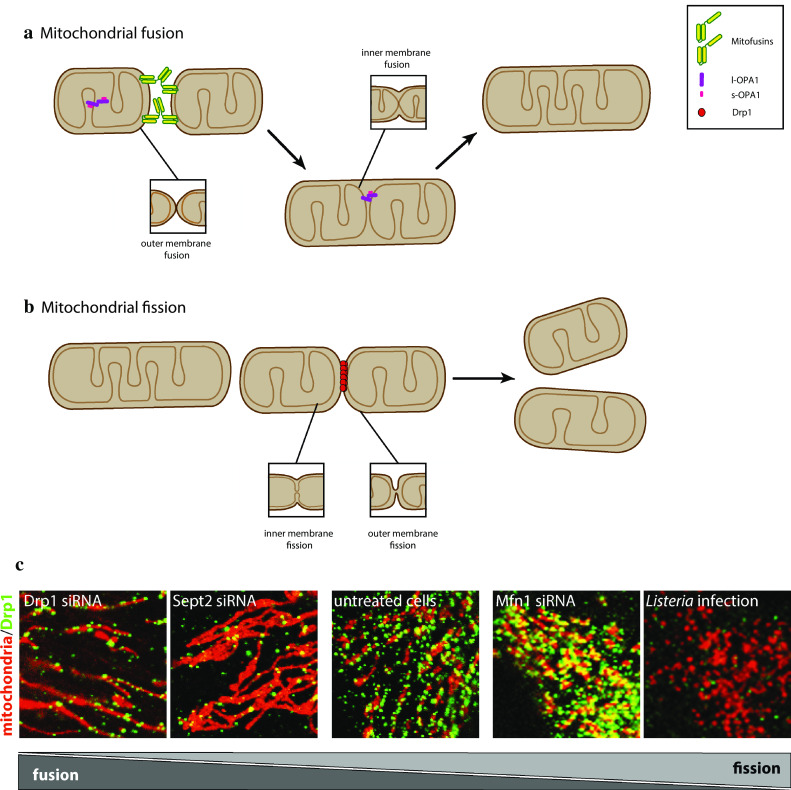
Mitochondrial fusion and fission. **a** Mitochondria tethering via homotypic (Mfn1–Mfn1 and Mfn2–Mfn2) and heterotypic (Mfn1–Mfn2) mitofusin interactions promotes OMM fusion, while inner membrane fusion is promoted by OPA1. **b** Schematic representation of mitochondrial fission. Enlargements of OMM and IMM fusion events that occur during mitochondrial fusion and fission. **c** Immunofluorescence showing changes in mitochondrial morphology (*red*) and Drp1 localization (*green*) in response to different perturbations. Adapted from [182, 190, 202]

**Fig. 2:**
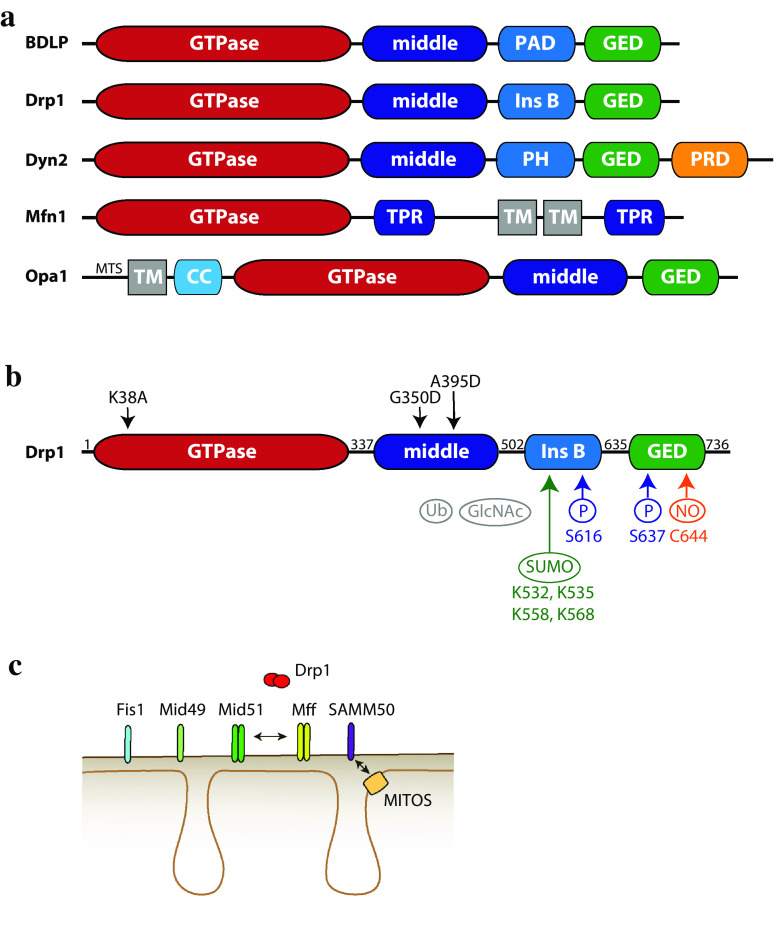
Domain structure of Drp1, its modifications and receptors. **a** The family of mitochondrial dynamics proteins and their bacterial ancestor, the bacterial dynamin-like protein BDLP. Abbreviations: paddle domain (PAD) and pleckstrin homology domain (PH), both responsible for lipid binding; proline-rich domain (PRD), tetratricopeptide (TPR) and coiled–coiled (CC) domains are involved in protein–protein interactions; GTPase effector domain (GED); insert B domain (Ins B); transmembrane domain (TM). **b** full-length human Drp1 (isoform 1, 736 aa). *Numbers* indicate the start of a new domain. Posttranslational modifications are *colour*-*coded*, and the modified amino acids have been indicated, except for ubiquitination and *O*-GlcNAcylation (*grey*), for which sites have not yet been identified. A few commonly used mutants are shown in *black*: K38A (dominant-negative), G350D (impaired in higher order oligomerization), A395D (natural mutation, impaired in tetramerization and higher order oligomerization). **c** Drp1 is recruited to the OMM via multiple transmembrane receptors. MiD51 and Mff are able to oligomerize and to interact with each other in the same fission foci. The SAMM50 protein has been shown to interact with the MITOS complex in the IMM

Mitochondrial fusion and fission often occur simultaneously and in a balanced manner within a cell, and an increase in either activity leads to hyperfused or fragmented mitochondria (Fig. 1). The complex membrane system of mitochondria is a challenge for fusion and fission and requires specialized molecular machineries on the OMM or IMM. It is interesting to note that the key proteins known to date to mediate mitochondrial fusion and fission are all nuclear-encoded, large GTPases, pointing to a common evolutionary origin (Fig. 2a). This may not be surprising, as despite differences in the membrane topology, both mitochondrial fission and fusion require the merging of membranes (Fig. 1a, b, inset), which is achieved through membrane remodeling proteins. Different model organisms including yeast, *Drosophila* and *C.elegans* have greatly contributed to our understanding of the molecular mechanisms that underlie mitochondrial fusion and fission. In this review, we will focus on mammalian cells, summarizing mitochondrial fusion and highlighting several recent discoveries that significantly expanded our knowledge on mitochondrial fission.

### Molecular basis and physiological roles of mitochondrial fusion

During mitochondrial fusion, the outer and inner membranes of two mitochondria fuse in a regulated manner. This permits content exchange and enables cross-complementation of mitochondrial DNA molecules, preventing the accumulation of mutant DNA in a specific mitochondrion [5, 6]. Although content exchange allows the mitochondrial network to adapt to the metabolic demand in a concerted manner (reviewed in [7]), it does not always lead to a completely homogeneous mitochondrial population within a single cell, and the mitochondrial membrane potential can vary among different mitochondria [8]. Mitochondrial fusion appears particularly important during stress and starvation conditions, where it is thought to maximize the efficiency of mitochondrial metabolism through the sharing of metabolites in the matrix (reviewed in [9]). Interestingly, although bacteria are not known to fuse, filamentous bacteria do exist (e.g. syncytial *Streptomyces* or septated filamentous cyanobacteria). Such filament formation can be induced by mutation in cell division proteins, or upon stress or nutritional changes, conditions potentially also encountered by the alphaproteobacterial mitochondrial ancestor.

In present-day mitochondria fusion is an active process that is mediated by Mitofusin 1 and Mitofusin 2 in human cells (Mfn1 and Mfn2, Fzo1 in yeast [10, 11]). These partially redundant large GTPases are embedded in the OMM, where they promote mitochondrial fusion (Fig. 1a). Mitofusins form hetero-oligomers, promote mitochondrial tethering similar to SNARE proteins and mediate GTP-dependent fusion [12]. Recently, it has been reported that mitofusins fold both in fusion-competent and fusion-incompetent conformations to regulate mitochondrial tethering [13]. An additional mechanism has been recently described for mitochondrial network formation, which would involve mitochondrial tubulation by kinesin KIF5B in concert with mitofusin-mediated fusion [14]. Recently, part of the Mfn1 structure has been solved and found to resemble that of bacterial dynamin-like proteins, uncovering an interesting evolutionary relationship [15, 16]. While Mfn1 has a higher GTPase activity and is ubiquitously expressed [17], Mfn2 displays tissue-specific expression [18] and has been linked to the type 2a subset of Charcot–Marie–Tooth disease, a group of hereditary peripheral neuropathies that can be caused by defects in several of the proteins regulating mitochondrial dynamics [19]. Interestingly, both mitofusins are essential for embryonic development in mice, where they play an important role in the maintenance of mitochondrial DNA (mtDNA) and oxidative phosphorylation [20, 21] and may also have additional protein-specific functions [22]. In addition, Mfn2 plays a role in ER–mitochondria tethering [23]. ER–mitochondria contact sites are crucial for interorganellar communication, including calcium signaling integration and lipid biogenesis [24, 25], and are emerging as an important player in mitochondrial fission (detailed below).

Although to a large extent coordinated, fusion of the OMM and IMM can occur sequentially, as suggested by the isolation of fusion intermediates in vitro and in vivo [26, 27]. This points to the presence of an IMM fusion machinery. A key molecule for IMM fusion is Optic Atrophy 1 (OPA1, Mgm1 in yeast), a three-membrane-pass protein that faces the intermembrane space (Fig. 1a) and is mutated in autosomal dominant optic atrophy [28, 29]. In contrast to Mitofusins, OPA1 does not need to be present on apposing membranes to mediate fusion [30]. In addition, OPA1 participates in the shaping of cristae, IMM invaginations whose remodeling plays an important role in the release of the proapoptotic mitochondrial inner membrane-associated protein cytochrome c during apoptosis [31]. OPA1 is highly regulated, both through differential splicing [32], and through proteolytic processing, which leads to long IMM-anchored OPA1 and short, soluble OPA1. The AAA-protease YME1L and the inner membrane metalloprotease OMA1 are involved in OPA1 processing [33]. Although both long and short OPA1 forms are found in OPA1 supercomplexes, long OPA1 appears to be required for fusion, while short OPA1 may play a role in IMM fission [34]. In mammals, a drop in the mitochondrial membrane potential and other stress factors block fusion and stimulate OPA1 processing by OMA1 [33]. OMA1 activation may, therefore, represent a key point of regulation, as its activation under stress conditions can result in complete conversion of long OPA1 to short OPA1, definitively preventing mitochondrial fusion until new OPA1 is synthesized. Although OPA1 regulation and function have been characterized in detail, further research is needed to uncover mechanistic details of the inner membrane fusion and fission processes, which may involve additional players.

### Mitochondrial fission: division for survival?

Mitochondrial fission divides the organelle into two often not equally sized daughter mitochondria (Fig. 1b). The best-characterized mitochondrial division machinery relies on dynamin-related protein 1 (Drp1/DNML1/DLVP/Dymple, Dnm1 in yeast), which is regulated at multiple levels. Knockout studies in mice showed that deletion of Drp1 is embryonically lethal, indicating that mitochondrial fission is an essential process [35, 36]. Tissue-specific knockout approaches and in vitro studies demonstrated that mitochondrial fission deficiencies result in respiratory defects [37]. At the cellular level, mitochondrial fission is important for organelle distribution during mitosis [38] and for proper distribution of mitochondria to neuronal synapses, where localized energy production supports synaptic functions [36, 39]. Interestingly, organellar and cellular quality control mechanisms have been linked to mitochondrial dynamics. At the organellar level, mitochondrial fission has been implicated in mitophagy, the autophagic process by which defective mitochondria are selectively degraded [40, 41]. Such defective mitochondria are characterized by a loss in the inner membrane potential, and are thereby recognized by the Pink1/Parkin system. Parkin then mediates ubiquitination of several OMM-associated proteins, which are then recognized by different ubiquitin-binding mitophagy receptors, targeting the whole mitochondrion for autophagic degradation (for recent reviews see [42, 43]). Limiting mitochondrial size through fission seems to be a prerequisite for mitochondria engulfment into autophagosomes [40, 44]. The extent to which Drp1 is an active player in mitophagy or not is, however, still debated [40, 43, 45–49].

In addition to mitophagy, mitochondrial fission has been shown to occur during apoptosis and to promote the release of the proapoptotic protein cytochrome c from the intermembrane space [50]. However, later studies indicated that apoptosis can proceed in Drp1-depleted cells [35, 36, 51, 52] and that fission does not necessarily correlate with apoptosis. In the following, we will summarize recent findings in mitochondrial fission, with a particular focus on the role of the cytoskeleton during the fission process.

## New insights into Drp1-dependent mitochondrial fission

### The dynamin-related protein Drp1

Dynamins are evolutionarily conserved, versatile molecules that are regulated by oligomerization and conformational changes to mediate membrane remodeling. A key protein in mitochondrial fission is Dynamin-related protein 1, which is conserved in opisthokonts (animals and fungi) and plants [53, 54]. Drp1 was first identified by several laboratories through the homology of its GTPase domain with that of endocytic dynamins [55–60]. Similar to endocytic dynamins, Drp1 multimerizes to form spirals on the mitochondrial outer membrane and hydrolyzes GTP, which cause a conformational change, enabling spiral compaction and resulting in mitochondrial constriction [61, 62]. Alternative splicing can produce up to eight different Drp1 isoforms, with cell type-specific expression patterns [59, 63, 64]. The best-characterized is isoform 3, which lacks 31 amino acids in the insert b domain [59, 63–66]. Drp1 has an apparent molecular weight of ~ 80kD and is composed of four domains (Fig. 2b): a GTPase domain (containing an insert A in the neuronal isoform), a middle domain, a variable domain (also called insert B), and a GTPase effector domain (GED), whose C-terminal coiled coil mediates multimerization [67]. The mechanochemical core of the protein comprises the GTPase, middle and GED domains and is sufficient to cause liposome constriction [60]. In contrast to dynamin, Drp1 lacks the pleckstrin homology domain that mediates binding to phosphoinositide-containing membranes, such as the inner leaflet of the plasma membrane (Fig. 2a). This may account for the fact that Drp1 does not localize to clathrin-coated pits [59] and its depletion/mutation does not affect the secretory pathway [68]. However, Drp1 was recently shown to play a role in endocytic vesicle formation in hippocampal neurons, suggesting that recruitment of Drp1 to different cellular structures may be regulated through cell type-specific mechanisms [69].

### Role of lipids in mitochondrial fission

For a long time thought to be only structural components of cellular membranes, lipids are now well recognized as active players in membrane remodeling. In addition to serving as recruiting or activating factors for proteins, lipids can affect membrane dynamics by lowering the energy barrier for fusion and fission, two energetically unfavorable processes. Here we briefly summarize the role of phospholipids in Drp1-mediated mitochondrial fission (see [70] for a comprehensive review on mitochondrial lipids).

The signature of mitochondrial membranes is the presence of cardiolipin (CL). CL is an atypical conical lipid and indirect evidence has implicated conical lipids in the generation of membrane curvature, which is required for both membrane fusion and fission [71]. CL is found primarily in the IMM, where it constitutes almost 20% of the total lipid content [72], but is also present in the OMM, in particular at IMM/OMM contact sites [73, 74]. Recent studies have shown that CL binds Drp1 at four lysines located in the variable domain [75]. Additionally, CL appears to recruit Drp1 to the OMM and stimulates the GTPase activity [76, 77]. This stimulation occurs only at concentrations matching those of IMM/OMM contact sites, suggesting that these specialized domains can serve as a platform for Drp1 recruitment and activation. Conversely, a recent report showed that Drp1 regulates CL clustering in liposomes, inducing a CL phase transition, which in turn triggers membrane constriction and fission [78]. An interesting recent report added a new layer of complexity to this picture, showing that mitochondrial phosphatidic acid (PA) can bind Drp1 and inhibit its GTPase activity [79]. PA is formed on the OMM by the action of mitochondrial phospholipase D (mitoPLD), a membrane-tethered enzyme, which utilizes CL as a substrate. The ability of Drp1 to interact with mitoPLD suggests that recruitment of the latter could be a way to balance Drp1 activation.

### Drp1-dependent mitochondrial fission as a multistep process

Surprisingly, Drp1, the key player of mitochondrial fission not only targets mitochondria, but also the ER [59, 80, 81] and peroxisomes [82]. This raises the question as to how differential targeting of Drp1 is achieved. Several studies showed that mitochondrial fission is a multistep process, in which specific targeting of Drp1 to the OMM relies on its numerous posttranslational modifications and on its transmembrane receptors in the OMM.

### Drp1 regulation by posttranslational modifications

#### Phosphorylation

Since mitochondria cannot be generated de novo, they have to be segregated into daughter cells during mitosis, which is achieved through extensive mitochondrial fission prior to mitosis. Mitochondrial network fragmentation coincides with phosphorylation of human Drp1 at S616 by Cdk1/cyclin B kinase, but the functional impact of this modification on Drp1 is currently unknown, as its GTPase activity does not seem affected in vitro. Interestingly, as cells exit mitosis, the mitochondrial network is restored via APC/C (anaphase-promoting complex/cyclosome)-mediated degradation of Drp1 [83]. Mitochondrial network fragmentation has been also observed upon phosphorylation of Drp1 S616 by Erk2 in cancer cells, where it appeared to play a role in tumor proliferation [84, 85]. Recently, Drp1 S616 has been found to be phosphorylated also by Cdk5 kinase in neurons, but its effect on Drp1 activity and mitochondrial network morphology is controversial [86, 87].

Drp1 S637 is phosphorylated by cyclic AMP-dependent protein kinase A (PKA) and this modification was found to inhibit the GTPase activity, most likely by preventing intra-molecular interactions between the GED and GTPase domain [88, 89]. This phosphorylation implicated Drp1 as a survival-promoting substrate of PKA, in particular during starvation, where elongation of the mitochondrial network and increased ATP production sustain metabolic needs [90]. Calcineurin was identified as the phosphatase responsible for S637 dephosphorylation [89] and was then shown to mediate calcium-induced, Drp1-dependent mitochondrial fission [91]. Calcineurin-mediated Drp1 dephosphorylation appeared to have a marginal effect on Drp1 oligomerization or its GTPase activity, while strongly regulating the recruitment of Drp1 to the OMM [91, 92]. Both phosphorylation sites are shown in Fig. 2b.

#### Ubiquitination

Ubiquitin is a 76-amino acid polypeptide, which is covalently attached to lysine residues of target proteins via an enzymatic reaction. Ubiquitin can either regulate protein stability by targeting modified substrates to proteasomal degradation or change their biological function by modulating functional interactions. Drp1 has been shown to be ubiquitinated by the ubiquitin ligase Parkin [93]. This modification targets Drp1 for proteasomal degradation and, therefore, Parkin depletion increased Drp1 levels and mitochondrial fragmentation. The OMM-anchored ubiquitin ligase MARCH5 (also known as MITOL) also plays a role in mitochondrial dynamics; however, it is not clear from the published reports whether MARCH5 specifically regulates fusion or fission [94–96]. Several proteins involved in mitochondrial dynamics are reported substrates of MARCH5, including Drp1, Mfn1, Mfn2 and Mid49 [97–100], possibly explaining the heterogeneity of published results concerning MARCH5 function. A recent report added another layer of complexity by showing that Drp1 is not only a substrate, but also a regulator of MARCH5 activity, along with Mff (Mitochondrial fission factor, a Drp1 receptor on the OMM, see below) [101]. New studies will shed light on the role of MARCH5 activity towards its several identified substrates.

#### Sumoylation

Small ubiquitin-like modifier (SUMO) is a ubiquitin-related protein, which can be covalently linked to a target protein. SUMOylation regulates protein conformation (and hence activity), localization and interaction with cellular partners [102]. The SUMO-conjugating enzyme Ubc9 and SUMO itself were identified as Drp1 interacting partners by yeast two-hybrid analysis and SUMO partially colocalized with Drp1 at fission sites [103, 104]. Later work identified MAPL (mitochondrial-anchored protein ligase) as the mitochondrial-anchored E3 SUMO ligase able to directly SUMOylate Drp1 in the variable domain [105, 106], Fig. 2b. Interestingly, four lysines are differentially spliced into three of the Drp1 isoforms, raising the possibility that SUMOylation might also account for functional differences among Drp1 isoforms [106]: SUMOylation led to increased Drp1 stabilization on the OMM and increased mitochondrial fission [104]. Drp1 SUMOylation is reversed by the SUMO protease SENP5 [107], in particular during mitosis, where Drp1 deSUMOylation correlated with increased binding and release of Drp1 to/from the OMM, thereby stimulating fission [108]. Phosphorylation and deSUMOyation thus appear to converge in activating Drp1 during mitosis.

#### S-nitrosylation

Nitric oxide (NO) is not only a neurotransmitter of the central nervous system: in addition to its signaling role, NO can be covalently linked to thiol groups of target proteins, such as cysteine residues, in a process called S-nitrosylation. S-nitrosylation can affect protein function, stability or subcellular location [109]. Drp1 was found to be S-nitrosylated on C644 (Fig. 2b) upon NO overproduction due to pathological conditions, such as accumulation of β-amyloid aggregates, key mediators of Alzheimer disease [110, 111]. This correlated with rapid Drp1-dependent mitochondrial fragmentation, followed by energy production failure and cell death [110]. However, these findings were subsequently questioned by Bossy et al. [112], who failed to detect a change in Drp1 oligomerization and GTPase activity upon S-nitrosylation. Additionally, OPA1 was also found to be S-nitrosylated. Therefore, at present S-nitrosylation of Drp1 may not be uniquely responsible for NO-induced mitochondrial fission.

#### *O*-GlcNAcylation

*O*-GlcNAcylation refers to the reversible but covalent attachment of N-acetyl-glucosamine to serine or threonine residues of a target protein. *O*-GlcNAcylation of Drp1 was shown to increase Drp1 GTP-binding activity, Drp1 translocation to the OMM and mitochondrial fission in cardiomyocytes [113]. *O*-GlcNAcylation of Drp1 was accompanied by decreased levels of Ser637 phosphorylation; further investigations are thus required to discriminate whether the effects observed upon *O*-GlcNAcylation are mediated by decreased Ser637 phosphorylation or whether *O*-GlcNAcylation directly affects Drp1 function. Recently, decreased *O*-GlcNAcylation levels were shown to result in an increase in Drp1-dependent mitochondrial fission, along with a decrease in the mitochondrial membrane potential and mitochondrial content [114]. An interesting aspect is that *O*-GlcNAcylation directly links mitochondrial morphology with the metabolic state of the cell, as the latter regulates cellular levels of the *O*-GlcNAc donor UDP-GlcNAc [115].

### Drp1 recruitment through receptors on the OMM: Mff, MiD49/51, and Fis1

Although being the master regulator of mitochondrial fission, more than 95% of Drp1 is cytosolic [68]. Hence, Drp1 recruitment to mitochondria is an early and key step in mitochondrial division, which is stimulated by the presence of four single-pass transmembrane Drp1 receptors that are anchored to the OMM: mitochondrial fission factor (Mff), the mitochondrial dynamics proteins 49 and 51 (MiD49 and MiD51) and Fis1.

#### Fis1

Fis1 was the first identified receptor for Drp1 [116, 117]. In yeast, Fis1 appeared to regulate mitochondrial morphology by recruiting the yeast Drp1 homologue Dnm1 to the OMM in concert with the partially redundant adaptor proteins Mdv1 and Caf4 [118, 119]. However, no Mdv1 and Caf4 homologs have been found in mammals, suggesting that the machineries for mitochondrial fission have diverged during evolution. In agreement with this hypothesis, to date there is mixed evidence about the role of Fis1 in mammals. Some reports have involved Fis1 in Drp1-dependent mitochondrial fission [120–122], and shown it binds Drp1 [120, 123]. These data were not reproduced in other studies [121, 124, 125]; furthermore, Fis1 knockout cells displayed very mild fission defects, raising questions about the precise role of Fis1 in Drp1 recruitment and mitochondrial fission [92, 123].

Fis1 was recently proposed to play a specific role in stress-induced mitochondrial fission [126, 127]. Indeed, induction of mitophagy caused Fis1 to form a complex with several ER proteins and Mff-recruited Drp1. Fis1 overexpression has in turn been shown to trigger mitophagy [40, 90, 128]. Fis1 deletion induces accumulation of the autophagy marker LC3 at mitochondria during stress-induced, Parkin-mediated mitophagy [127, 129] and Fis1 knockout cells show severely impaired mitophagy [128]. Thus, the role of Fis1 in mitochondrial dynamics may be restricted to specific physiological conditions such as apoptosis and mitophagy.

#### Mff

Mff is a 30-kDa C-tail-anchored membrane protein, which is considered a prime Drp1 receptor at both mitochondria and peroxisomes, as for both organelles its overexpression stimulates fission and Drp1 recruitment, while its ablation induces elongation [123, 130]. Unlike Fis1, Mff accumulates in discrete foci, where it recruits dimeric or higher order complexes of Drp1 [131]. Interestingly, the formation of Mff clusters is lost in Drp1 knockout cells, suggesting that Drp1 binding in turn influences Mff oligomerization [132]. Mff might promote Drp1 recruitment to facilitate severing of mitochondria specifically at ER-mitochondria contact sites, as suggested by imaging experiments and by mutagenesis data on Drp1 [133, 134]. The regulation of Drp1 by Mff is complicated by the presence of several Mff isoforms [130], which were recently shown to have a differential effect on the Drp1 isoforms [77]. Additionally, Mff also appears to increase the Drp1 GTPase activity and may thereby promote Drp1 spiral compaction and consequently the severing of mitochondria [131, 135]. Interestingly, Mff appears to be restricted to metazoans, highlighting that the overall conserved mitochondrial fission machinery can accommodate additional proteins, likely providing new ways to regulate fission.

#### MiD49/MiD51

Evolutionarily, mitochondrial division (MiD) proteins appeared after Mff, since they are only present in chordates, possibly reflecting a higher specialization in the mitochondrial division machinery. MiDs were unambiguously identified as Drp1 receptors through immunoprecipitation and yeast two-hybrid assays [124]. As in the case of Mff [123], the interaction between Drp1 and MiDs also appears to be transient, requiring chemical crosslinking for its detection [124]. MiDs bear an N-terminal transmembrane domain that anchors them to the OMM, and are not present on peroxisomes. The structures of the MiD49- and MiD51-soluble domains have been recently solved, revealing a similar nucleotidyltransferase domain [136–138]. Despite this similarity, MiD49 and MiD51 differ in their nucleotide-binding capacity, as MiD49 lacks the critical residues required for nucleotide binding. Interestingly, a MiD51 mutant deficient in nucleotide binding was still capable to recruit Drp1 to mitochondria, but appeared unable to promote mitochondrial fission [137].

#### Cooperation or specialization?

Why does Drp1 need several receptors to localize to mitochondria? Although the receptors may play independent and partially redundant functions in Drp1 recruitment [92, 125, 132], recent work showed that Mff and MiDs colocalize with the ER at mitochondrial fission sites, suggesting functional cooperation of the receptors [139]. Given that Mff preferentially interacts with dimeric Drp1 and stimulates Drp1 GTPase activity [65, 131], while MiD51 inhibits it [125, 140], a plausible speculation might be that Mff first recruits dimeric Drp1 at mitochondria and MiDs would then promote Drp1 self-assembly, while inhibiting its GTPase activity. Finally, Mff would stimulate the GTPase activity of the assembled Drp1 oligomer to promote fission. The canonical Drp1 receptors Mff and MiD49/51 may thus cooperate under specific conditions or in specific cell types. The picture has been recently further complicated by additional OMM proteins shown to also promote Drp1 recruitment, such as SAMM50, a component of the SAM complex, which inserts beta barrels in the OMM [141]. SAMM50 has been shown to interact with Drp1 and induce Drp1-dependent mitochondrial fission through an unknown mechanism [142]. Interestingly, SAMM50 could coordinate outer and inner membrane fission, as it also interacts with VDAC, a component of OMM/IMM contact sites and with the MINOS/MITOS complex in the IMM.

### Determining the site of fission along a mitochondrion

#### Initial constriction occurs at ER–mitochondrial contact sites

ER–mitochondria contact sites were described already in the 1960s [143] and are visible by EM, but the molecular nature of the proteinaceous tether between the two organelles is still largely obscure. In mammalian cells, Mfn2 is thought to tether the ER and mitochondria [23, 144] and ER–mitochondrial contacts were also found to contain a complex of the IP3 receptor, the chaperone Grp75 and the voltage-gated anion channel VDAC [145]. Additional proteins found at contact sites include Rab32 [146], mammalian target of rapamycin complex 2 (mTORC2, [147], PACS2 [148]), and the recently identified Syntaxin 17, an evolutionarily conserved SNARE protein, a possibly ancestral tethering molecule [149]. ER–mitochondria contact sites can span up to 20% of the mitochondrial surface [150] and were mainly thought to serve as a platform for lipid exchange and Ca^2+^ signaling [24, 25]. In a spearheading work, Friedman and colleagues showed that ER–mitochondria contact sites also play a role in mitochondrial division: 88% of the mitochondrial fission events were found to be marked by ER tubules, which wrapped around mitochondria, and appeared to promote fission by constricting mitochondria in a process termed ER-associated mitochondrial division (ERMD) [133]. Intriguingly, the ER seems to play an analogous role in the fission of endosomes [151]. ER-marked mitochondrial constrictions show a diameter comparable to that of Drp1 helices and may, therefore, physically facilitate Drp1 recruitment or be actively sensed by Drp1. The ability of dynamin and dynamin-like proteins to detect membrane deformations [152], polymerize, and induce local membrane rearrangements seems to be ancestral, as a bacterial dynamin-like protein has recently been shown to accumulate in foci of antibiotic or phage-induced membrane deformations to protect membrane integrity [153]. The ER could also induce mitochondrial fission by promoting lipid insertion at contact sites or by clustering Mff and MiD49/51 [133, 139], which is expected to promote Drp1 recruitment. In addition, proteins present at ER-induced preconstrictions may support local Drp1 activation, as shown for MiD51 [154] and Syntaxin 17, which has been recently shown to specifically bind GTP-loaded Drp1 [149].

#### ER-associated mitochondrial division preferentially occurs next to the nucleoid

Mitochondria are the only organelles of animal cells that harbour their own circular genome (mtDNA), which associates with proteins that pack it into a nucleoid. As for the nuclear genome, the distribution of mtDNA molecules to daughter mitochondria is a crucial event. Early studies noticed that nucleoids are often localize at the tips of mitochondria [155, 156] or next to Drp1-marked constrictions [157], suggesting a link to mitochondrial fission. Ban-Ishihara then found that mitochondrial fission occurs in the vicinity of a nucleoid in 70% of all cases, and nucleoids cluster in Drp1-deficient cells [158]. This confirmed previous work showing that mitochondrial fission prevents nucleoid clustering and mtDNA loss in cellulo, later also shown in vivo [159, 160]. Interestingly, the nucleoid position correlated with ER–mitochondria contact sites in 85% of cases, raising the unsolved question as to how the nucleoid position is sensed by the ER across the two mitochondrial membranes and vice versa. Not only do nucleoids seem to communicate their position to the ER across the IMM and OMM, but recent data showed that the number of ERMD events increases up to threefold in presence of replicating nucleoids [161], highlighting the importance of the functional state of the nucleoid. mtDNA replication was found to occur upstream of Drp1 recruitment and mitochondrial division, and blocking nucleoid replication had been previously shown to cause Drp1 and Mff redistribution [158]. Whether ERMD at replicating nucleoids equally distributes the newly synthesized mtDNA into daughter mitochondria appears controversial [158, 161].

### The role of the cytoskeleton in Drp1-dependent fission

#### Actin and actin-binding proteins

The first hint that actin may be involved in mitochondrial dynamics came from an early study in which actin depolymerization by cytochalasin D was shown to slow down mitochondrial fusion in mammalian cells [162]. In a pioneering work, De Vos and colleagues subsequently showed that actin depolymerization by cytochalasin D or latrunculin A impairs CCCP-induced mitochondrial fission by preventing Drp1 recruitment to mitochondria ([163], see Table 1 for an overview of drugs commonly used to manipulate mitochondrial dynamics). Stabilization of actin filaments through Wiskott–Aldrich Syndrome protein (WASP) overexpression was shown to increase mitochondrial length in *Drosophila* neurons, impairing Drp1 recruitment and eventually leading to neurodegeneration [164]. In search for factors mediating the actin-dependent localization of Drp1 to mitochondria, Myosin II was identified as a crucial player, a finding that the authors confirmed in mammalian cells.

**Table 1:** Most commonly used treatments to manipulate mitochondrial morphology

Treatment	Effect on mitochondrial morphology	Mechanism of action	References
CCCP	Fragmentation	Protonophore	[163]
FCCP	Fragmentation	Protonophore	[91]
Oligomycin	Fragmentation	Mitochondrial complex V inhibitor	[163]
Ionomycin	Fragmentation	Mitochondrial Ca^2+^ ionophore	[135]
BAPTA-AM	Fragmentation	Ca^2+^ chelator	[133]
High extracellular K^+^	Fragmentation	Mitochondrial Ca^2+^ influx	[211]
KN93	Fragmentation	CamKII inhibitor	[211]
UO126	Fragmentation	MAPK–ERK inhibitor	[211]
Arachidonic acid	fragmentation	Permeability transition pore inducer	[91]
mdivi-1	Hyperfusion	Inhibition of yeast Dnm1 Mitochondrial complex I assembly inhibitor	[212, 213]
Latrunculin B	Hyperfusion	Actin depolymerization	[166]
Cycloheximide	Hyperfusion	Translation inhibitor	[214]

The mechanism by which actin polymerization at mitochondria would regulate Drp1 recruitment has been recently investigated in more detail. Ji et al. showed that actin polymerization on mitochondria supports Drp1 maturation by incorporation of small Drp1 oligomers already associated with mitochondria. Interestingly, also MiD-containing complexes have been shown to coalesce and mature, raising the possibility that MiD foci maturation might also be dependent on actin dynamics [139]. Actin polymerization on mitochondria was found to precede the appearance of large Drp1 oligomers at fission sites upon ionomycin-induced mitochondrial fission [135]. In vitro studies showed that actin directly binds Drp1 in a GTP-dependent manner, stimulating its GTPase activity, which is further increased by the soluble domain of Mff [135, 165].

The identification of the ER as a major player in regulating mitochondrial pre-constriction upstream of Drp1 has set the stage for the discovery of the role played by the ER-localized form of inverted formin 2 (INF2) [166]. Together with the Arp2/3 complex and tandem monomer-binding proteins (TMBPs), formins constitute one of the three actin nucleator families characterized to date (reviewed in [167]). Interestingly, INF2 mutations have been associated with a subset of Charcot–Marie–Tooth disease [168], a disease in which several other genes regulating mitochondrial morphology have also been implicated, such as Mfn2, GDAP1 or DNML2 (https://ghr.nlm.nih.gov/condition/charcot-marie-tooth-disease). INF2 is a differentially spliced protein, which promotes the polymerization of linear actin filaments [169]. Depletion of the ER-targeted form of INF2 was found to increase the mitochondrial length and reduce Drp1 localization at mitochondria. Recently, Manor et al. identified Spire1C as a new player in INF2-dependent mitochondrial fission [170]. Spire1C, a splicing isoform of Spire1, localizes to the OMM and belongs to the TMBP actin nucleator proteins. TMBPs bind the pointed end of the actin filament, leaving the fast-growing barbed end free to associate with formins that stimulate further growth [171]. Thus, the discovery of Spire1C may explain why INF2 induced actin polymerization specifically at ER-mitochondria contact sites despite displaying an even ER membrane staining [169]. In addition, pull-down assays revealed a direct interaction between the soluble domains of Spire1C and INF2. Importantly, inhibition of the Spire1C–INF2 interaction decreases ER–mitochondria overlaps and mitochondrial fission [170]. Spire1C overexpression induces actin polymerization on mitochondria and a significant increase in fission, while its downregulation had the opposite effect, inducing mitochondrial elongation. Further work is needed to uncover the signals that regulate the interaction between Spire1C and INF2.

**Fig. 3:**
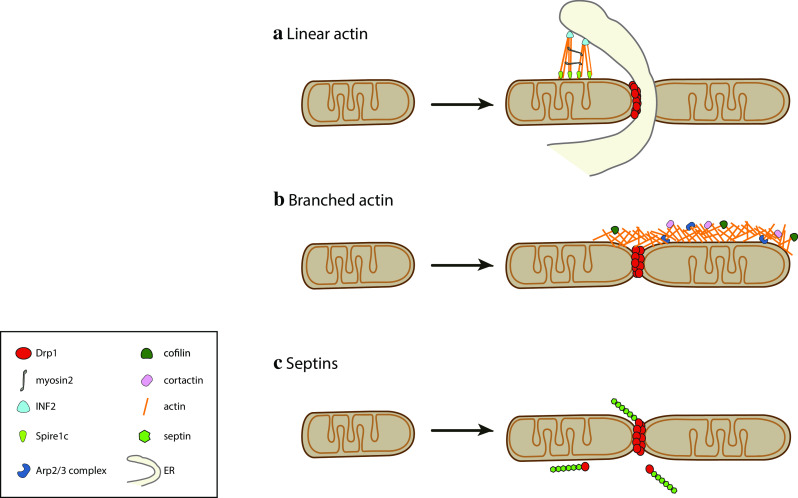
The cytoskeleton contributes to Drp1-dependent mitochondrial fission. **a** Linear actin polymerized by INF2 and Spire1C at the ER–mitochondria contact sites contributes to mitochondrial constriction with the aid of Myosin II, promoting Drp1 recruitment and mitochondrial fission. **b** Arp2/3 complex-mediated polymerization of branched actin on the OMM regulates Drp1 dynamics and mitochondrial fission. **c** Septins interact with Drp1 to regulate Drp1-mediated mitochondrial fission

How does INF2-induced actin polymerization provide the force required for mitochondrial (pre)constriction? Higgs and collaborators recently identified Myosin II as a downstream effector in INF2-dependent mitochondrial division [172]. Myosin II was found at mitochondrial fission sites in an actin- and INF2-dependent manner and its depletion or chemical inhibition decreased mitochondrial fission rates and mitochondria-associated Drp1 levels [135, 172]. These findings support a model in which the INF2-mediated polymerization of antiparallel actin bundles at the ER-mitochondria contact sites leads to recruitment of Myosin II, whose activation by an as yet unidentified signal would promote actin filament pulling, resulting in mitochondrial constriction (Fig. 3a). This process has been termed mitokinesis, in analogy to cytokinesis and to the term mitochondriokinesis introduced by Kuroiwa [173]. Of note, Myosin II has already been described to regulate membrane fission in other cellular pathways such as cytokinesis [174], phagocytosis [175] and *trans*-Golgi fission [176].

Recent work identified transient actin structures on the OMM that are not limited to ER–mitochondria contact sites [135] (see also Fig. 3b). Interestingly, impairment of Drp1-dependent fission was associated with an accumulation of F-actin on the OMM, suggesting that actin disassembly requires Drp1 activity. The authors identified Arp2/3-dependent actin polymerization as an additional actin polymerization mode that participates to mitochondrial fission [177]. The Arp2/3 complex [178] differs from formins and TMBPs in that it creates branched actin structures. Intriguingly, depletion of Arp2/3, cofilin and cortactin impaired FCCP-induced fission, while resulting in the accumulation of Drp1 oligomers on highly elongated mitochondria. These oligomers were hypothesized to represent non-functional Drp1 fission complexes, similar to what has been observed upon MiD49/51 overexpression, which leads to mitochondrial elongation with a concomitant accumulation of actin and non-functional Drp1 on the OMM [124].

Given that the Arp2/3 complex is involved in actin polymerization at several subcellular locations [167], an interesting open question is how differential regulation is achieved. Recent data on different models showed that the composition of the Arp2/3 complex is variable; while Arp2 and Arp3 are invariant and essential core subunits, the other subunits (ArpC1–ArpC5) play a regulatory role, and their substitution or posttranslational modification appears to determine localization and activity of the complex (reviewed in [179]). Future work will elucidate the exact composition of the Arp2/3 complex acting in mitochondrial fission, and its associated molecules, including the nucleation promoting factors that activate it.

A detailed description of the F-actin dynamics on the entire mitochondrial network in cultured HeLa cells showed that a wave of transient actin polymerization forms on a subset of mitochondria, rapidly cycling through the entire mitochondrial network and locally coinciding with fission [180]. These cyclic waves of actin-induced mitochondrial fission were proposed to regulate mitochondrial morphology at the level of the whole mitochondrial network. Actin appeared to assemble preferentially on elongated mitochondria, promoting their fragmentation, then promptly disassembled to allow refusion. Drug inhibition experiments showed that this process depends on Arp2/3 and formins, confirming the involvement of both actin polymerizing factors in actin-driven mitochondrial fission. Indeed, INF2/Spire1C- and Arp2/3-mediated actin polymerization may be to some extent functionally redundant, allowing Arp2/3-mediated actin polymerization to take over in cells that express low levels of INF2, such as epithelial or immune cells [181]. This would implicate that according to the tissue, different types of actin structures (linear versus branched) can fulfil a similar function in mitochondrial division. We currently lack a clear view of the actin filament structure during mitochondrial fission, a comprehensive list of the associated components, as well as the mechanisms that stimulate it beyond uncouplers [163, 177] and increases in intracellular calcium [135, 182].

Targeted actin polymerization participates in membrane fission in a number of different processes, such as endocytosis [183] and endosome fission [184], where it might act in conjunction with the ER [151]. An intriguing parallel can also be drawn with the intracellular bacterium *Listeria monocytogenes*, which initiates actin polymerization at the bacterial surface through the surface protein ActA [185]. This bacterial-induced local actin polymerization has been shown to compensate for a fission defect caused by deletion of a bacterial murein hydrolase [186] and also to regulate the timing of the division cycle of wild-type *Listeria* inside cells [187]. Whether the mechanisms by which actin stimulates this plethora of fission processes are the same or not remains an open question.

#### Septins

In contrast to actin, septins have been implicated in mitochondrial fission only very recently. Septins are a family of GTP-binding proteins discovered more than 40 years ago as regulators of cytokinesis in yeast [188, 189]. In humans there are 13 septin-coding genes, with many of them being expressed in a tissue-specific pattern. Now fully accepted as components of the cytoskeleton, septins can heterooligomerize into non-polar filaments, which can then assemble into higher order structures such as rings, bundles and gauzes. At present septins have been involved in diverse cellular processes such as cytokinesis, ciliogenesis, axon guidance and endocytosis and are thought to restrict protein diffusion at membranous structures such as the cell cortex, the yeast bud neck, and the ER, as well as restricting bacterial actin-based motility [188, 189].

A recent study highlighted a new role for septins as regulators of Drp1-dependent mitochondrial fission [190] (Fig. 3c). Sept2 was found to interact directly with Drp1, and depletion of Sept2 or Sept7 increased mitochondrial length. Sept2 depletion also delayed FCCP-induced mitochondrial fission and looping. About 30% of the mitochondrial constrictions appeared to be positive for Sept2, indicating that it might transiently associate with fission sites or be involved in a subset of fission events, as it has been proposed for INF2 and Myosin II [191]. What is the functional role of the Sept2–Drp1 interaction? Imaging coupled to mitochondrial fractionation experiments revealed that Sept2 is important for efficient recruitment of Drp1 to mitochondria, as Sept2-depleted cells displayed less Drp1 on mitochondria, similar to INF2 and Myosin II depletion [166, 172] (Fig. 2c). Septins might facilitate Drp1 recruitment through multiple mechanisms: first, septins might directly promote mitochondrial pre-constriction and Drp1 retention. Second, it is tempting to speculate that septins function as a scaffold at mitochondria and promote functional interactions of Drp1 with other proteins involved in mitochondrial fission, similar to the function that septins have already been shown to fulfil in other contexts, such as cytokinesis [192]. For example, septins might promote posttranslational modifications of Drp1 that allow its retention onto mitochondria. Multiple septins have indeed been shown to interact with the SUMOylation machinery by yeast two-hybrid screening and could, therefore, act as a scaffold to promote Drp1 SUMOylation. Another way septins might promote Drp1 function is by affecting its oligomerization status on mitochondria, similar to actin [135]. In this context, Ji et al. noticed cytosolic Drp1 oligomers, which may be associated with cytoskeletal structures. In agreement with this, we observed septin-associated oligomeric Drp1 structures in the cytosol (our unpublished observation), while Strack et al. found microtubule association of a specific Drp1 isoform, Drp1-x01 [66]. Different cytoskeletal structures may hence act as a cytosolic reservoir for delivering Drp1 oligomers to mitochondria.

#### Dynamin performs the terminal abscission step

**Fig. 4:**
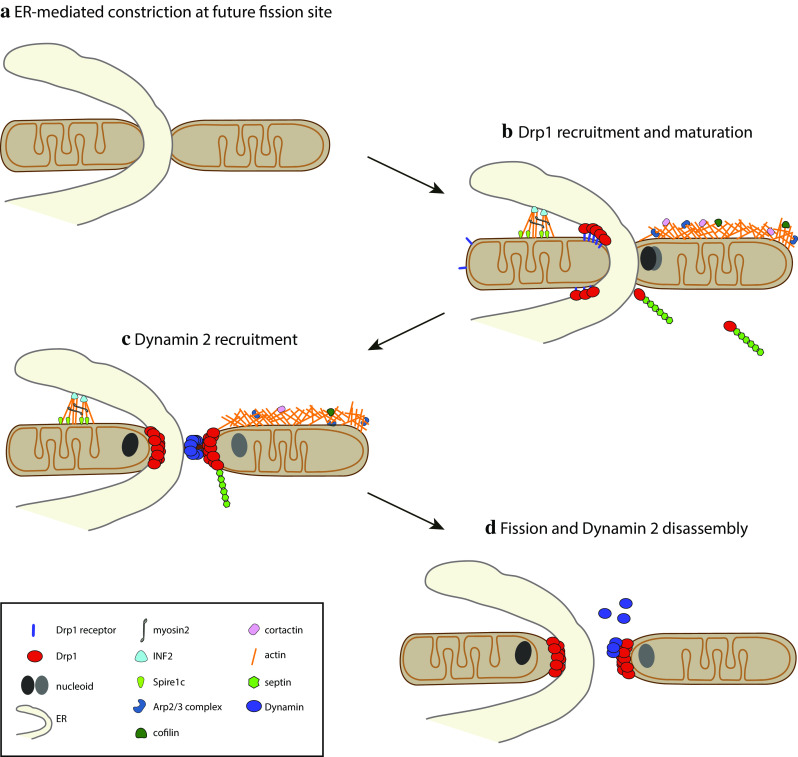
Mitochondrial fission is a multistep process. **a** The site of fission on mitochondria is first marked by an ER tubule which constricts mitochondria by wrapping around them. **b** Drp1 is recruited to the OMM with the aid of receptors, actin and septins, oligomerizes and initiates mitochondrial constriction. **c** Dynamin is recruited through an unknown mechanism and **d** drives mitochondrial abscission upon GTP hydrolysis

In vitro studies have shown that Drp1 is able to induce liposome tubulation and branching [60], but no fission has been observed in contrast to what has been observed with the endocytic Dynamin 1, which tubulates and severs liposomes in a GTP-dependent fashion [193]. Lee et al. have recently solved this conundrum by showing that the ubiquitous endocytic Dynamin 2 (Dyn2) terminates mitochondrial abscission downstream of Drp1 [194] (Fig. 4). Dyn2 required both its GTPase activity, as well as its PH and polyproline lipid-binding domains to mediate mitochondrial fission, although the structural arrangement of these domains with respect to the OMM remains unclear. Strikingly, Dyn2 mutations have been found associated with Charcot–Marie–Tooth type 2 disease and a recent case report showed that the Dyn2 R369W mutation induced multiple mtDNA deletions [195], reinforcing the link between mitochondrial dynamics and mtDNA maintenance. Interestingly, peroxisome fission has also been shown to require a cooperation between the mitochondrial dynamin Dnm1p and the endocytic dynamin Vps1p in yeast [196]. In mammalian cells, the ER, Arp2/3, the actomyosin system and Dyn2 [184] (Fig. 4) are all implicated in both endosomal and mitochondrial fission, raising the question as to how specific targeting is achieved [151, 184, 196, 197].

From an evolutionary perspective, it is interesting to note that an implication for endocytic dynamins in mitochondrial fission has been also shown in eukaryotes as distinct as the red algae *Cyanidioschyzon merolae* and the parasite *Trypanosoma brucei* [198, 199]. Consistent with the partnership between Dyn2 and Drp1 uncovered by Lee et al., a recent study proposed that the evolutionary ancestor of endocytic dynamins and Drp1 mediated both mitochondrial and vesicle abscission [53].

In contrast to Drp1, Dyn2 seems to associate only very briefly with mitochondrial division sites and segregates asymmetrically, suggesting important differences in the assembly and disassembly kinetics of Drp1 and Dyn2 [194]. Like endocytic dynamins, Drp1 disassembly requires GTP hydrolysis [60] and in addition possibly calcium [194], but although advances have been made by structure–function studies [200, 201], relatively little is known concerning the regulation of this essential step of the fission cycle.

### Drp1-independent fission

The canonical mitochondrial fission process occurs through Drp1 (Fig. 3); however, accumulating evidence suggests the presence of Drp1-independent fission mechanisms. For instance, during *L. monocytogenes* infection of epithelial cells, the mitochondrial network undergoes a dramatic fragmentation with concomitant impairment of the respiration capacity [182]. Surprisingly, Drp1 oligomers are absent from fragmented mitochondria (Fig. 1c). Analysis of this phenomenon in Drp1-depleted cells as well as in Drp1KO MEF revealed that *L. monocytogenes*-induced mitochondrial fragmentation is Drp1 independent [202]. Another example is provided by the abscission of mitochondria-derived vesicles (MDVs) [203, 204]. MDVs are induced by oxidative stress in a Parkin and Pink1-dependent manner, bud off mitochondria in a Drp1-independent manner, and then deliver their content to the lysosome to allow the selective removal of damaged proteins from the OMM [204].

Recently, mitophagy was reported to also occur in the absence of Drp1 [48]. Through live cell imaging experiments, the authors showed that upon induction of mitophagy, a mitochondrial bud emerges and is progressively isolated from the parental mitochondrion to finally detach by a still unidentified mechanism, which does not rely on Drp1. It is interesting to note that while Drp1 is required for embryonic development, it appears to be dispensable for the viability of in vitro-cultured cells [35]. This further suggests the existence of Drp1-independent mitochondrial fission mechanisms with a basal activity, which can be boosted under specific conditions such as oxidative stress, mitophagy induction or *Listeria* infection.

### Open questions

Although the appearance of the mitochondrial fission machinery has been postulated to precede that of fusion in evolution [173], no conserved machinery has been identified that would mediate fission of the inner mitochondrial membrane. A bacterial-derived system which induces fission from the matrix through an FtsZ homolog was detected across multiple eukaryotic species, suggesting that it was an ancestral mode of division [54], but little is known in mammalian cells. Because mammals lack a recognizable homolog of the bacterial fission protein FtsZ [53, 54] and the appearance of the organelle-dedicated dynamin Drp1 through gene duplication was found to coincide with the loss of FtsZ [53], Drp1 is currently thought to simultaneously sever both the OMM and the IMM. In agreement with this hypothesis, activation of Drp1 results in a fragmented mitochondrial network. But matrix constrictions have been observed in the absence of functional Drp1 in mammalian cells, suggesting the presence of an inner membrane fission machinery [205] which would respond to uncouplers or other stimuli that induce fission by acting directly on mitochondria. A few candidates for IMM fission have been proposed, including the inner membrane proteins TMEM11 [206] and MTGM/Romo1 [207], intermembrane space proteins as MTP18 [208] and the short, soluble form of OPA1 [209]. While overexpression of these candidates induced mitochondrial fission, their depletion did not always yield the hyperfusion phenotype that would be intuitively expected. We anticipate that the model for mitochondrial fission will be further refined by integrating the characterization of new players with the advent of new imaging techniques that allow high-resolution imaging of mitochondria with limited photodamage [135, 210].

### Electronic supplementary material

Below is the link to the electronic supplementary material.

Supplementary material 1 (TIFF 1884 kb)
