# Supplementary figures and images for: The ever-growing complexity of the mitochondrial fission machinery
Source: Cell Mol Life Sci. 2017 Aug 5;75(3):355–74. doi: 10.1007/s00018-017-2603-0 (PMC5765209; doi:10.1007/s00018-017-2603-0)

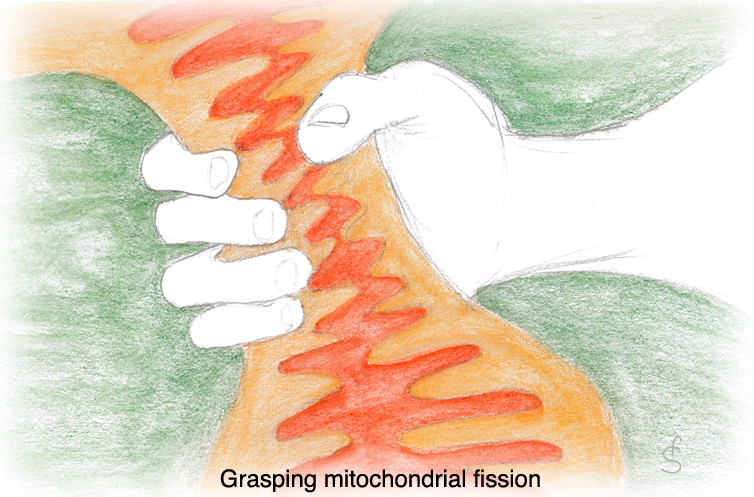

Supplement: Supplementary file 1 — Supplementary material 1 (TIFF 1884 kb) [file 18_2017_2603_MOESM1_ESM.tif]
